# Anterior Chamber Angle Changes and Its Associated Factor After Posterior Chamber Phakic Intraocular Lens Implantation in Eyes With Shallow Anterior Chambers

**DOI:** 10.1167/tvst.14.8.41

**Published:** 2025-08-28

**Authors:** Shengtao Liu, Fang Liu, Mingrui Cheng, Chiwen Cheng, Xiaoying Wang, Xingtao Zhou

**Affiliations:** 1 Department of Ophthalmology and Optometry, Eye and ENT Hospital, Fudan University, Shanghai, People's Republic of China; 2 NHC Key Laboratory of Myopia (Fudan University), Key Laboratory of Myopia, Chinese Academy of Medical Sciences, Shanghai, People's Republic of China; 3 Shanghai Research Center of Ophthalmology and Optometry, Shanghai, People's Republic of China; 4 The Second Affiliated Hospital, Jiangxi Medical College, Nanchang University, Nanchang, People's Republic of China

**Keywords:** anterior chamber angle, anterior chamber depth, implantable collamer lens, vault

## Abstract

**Purpose:**

The purpose of this study was to investigate anterior chamber angle (ACA) changes after implantable collamer lens (ICL) placement in eyes with shallow anterior chamber depth (ACD) and the effect of vault size on ACA changes.

**Methods:**

In this prospective cohort study of 120 patients (120 eyes) undergoing ICL implantation, participants were stratified into shallow (ACD ≤3.0 mm, *n* = 60) and normal ACD groups (ACD >3.0 mm, *n* = 60). Trabecular-iris angle 500 (TIA_500_), angle opening distance 500 (AOD_500_), trabecular-iris space area (TISA_500_), angle opening distance circumference area 500 (AODA_500_), trabecular-iris circumference volume 500 (TICV_500_), and anterior chamber volume (ACV) measured by swept source optical coherence tomography (SS-OCT) preoperatively and at 3 months postoperatively were compared within and between groups and according to vault size.

**Results:**

Relative to the preoperative values, the TIA_500_, AOD_500_, and TISA_500_ values decreased significantly in both groups (all *P* < 0.01). Similarly, the AODA_500_, TICV_500_, and ACV values decreased by 52.1%, 48.2%, and 38.9%, respectively, in the shallow ACD group (all *P* < 0.001), and by 59.2%, 56.6%, and 37.4%, respectively, in the normal ACD group (all *P* < 0.001). However, the reduction in all ACA parameters in the shallow ACD group in each meridian was significantly lower than that in the normal ACD group, both for low (all *P* ≤ 0.005) and high vaults (all *P* ≤ 0.004). Correlation analyses indicated that vault size was positively correlated with the decrease in ACA parameters in both groups (all *P* < 0.05).

**Conclusions:**

Anterior segment structure was more crowded in patients with a shallow ACD. This necessitates strict ICL size limits (≤12.2 mm for ACD <2.8 mm) and prioritizes long-term SS-OCT surveillance of the superior-nasal quadrant, particularly in high-vault cases.

**Translational Relevance:**

For patients with a shallow anterior chamber implanted ICL, placement in the superior-nasal quadrant should be considered.

## Introduction

The Visian implantable collamer lens (ICL; STAAR Surgical AG, Nidau, Switzerland) is a posterior chamber phakic intraocular lens that is placed on the ciliary sulcus. Although its efficacy in myopia correction has been widely recognized, it has also been associated with postoperative vault-related complications.^1^^–^^3^ Anterior chamber depth (ACD) is the primary parameter for screening suitable patients for ICL implantation. In the European Union, an ACD greater that 2.8 mm is required,^4^ whereas the US Food and Drug Administration (FDA) recommends an ACD of at least 3.0 mm to avoid increased rates of vault-related complications.^5^ Alfonso et al. reported that the ACD is significantly shallower in eyes with acute intraocular hypertension after ICL surgery.^6^ Therefore, the changes in the anterior chamber angle (ACA) in patients with a shallow anterior chamber warrants further investigation.

A previous study showed that the postoperative ACA was significantly associated with the preoperative ACA and vault size, and proposed that the vault was the main influencing factor of ACA alteration.^7^^,^^8^ For patients with shallow anterior chambers, determining the appropriate ICL size to obtain a suitable vault may be more difficult. Due to the small anterior chamber volume (ACV) and narrow space, the postoperative vault tends to be lower than the expected, increasing the risk of developing cataract.^5^^,^^9^ Conversely, significant angle narrowing and goniosynechia is also a risk after ICL implantation.^7^^,^^8^ Therefore, it is significant to evaluate the impact of different vault sizes on the change of ACA after ICL implantation in patients with shallow anterior chambers.

At present, swept source optical coherence tomography (SS-OCT; CASIA2, Tomey, Nagoya, Japan) can obtain the circumferential ACA parameters and provide accurate automatic three-dimensional quantification of the anterior segment, which can be used to evaluate the ACA after ICL surgery.^10^ Based on the above, the aim of this study was to assess the effect of vault size on ACA changes in patients with a shallow ACD. In this study, we evaluated and compared the circumferential ACA parameters under different vault range between patients with shallow and normal anterior chambers after ICL implantation.

## Methods

### Study Design and Population

**Figure 1.**
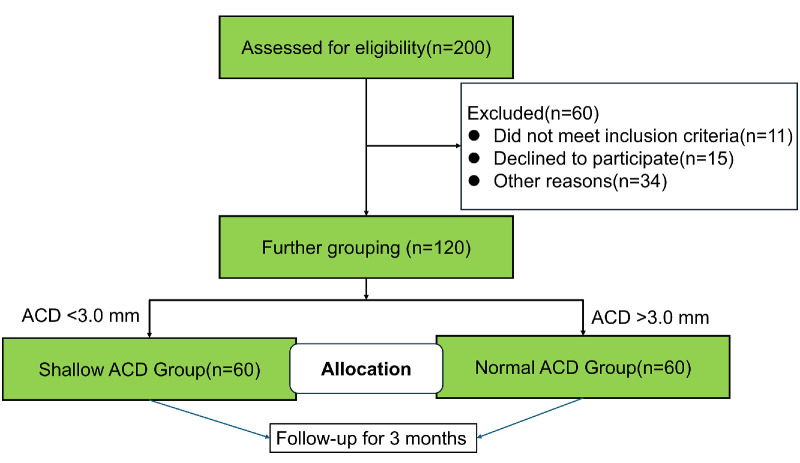
Flow chart of patient recruitment.

This prospective cohort study (Fig. 1) included 120 patients (120 eyes) who underwent ICL V4c implantation from May to November 2022 at the Eye and ENT Hospital, Fudan University (Shanghai, China), and study was approved by the Ethics Committee of the Fudan University Eye, Ear, Nose, and Throat Hospital (2021010), and all procedures were in accordance with the Declaration of Helsinki. All patients provided written informed consent prior to the examinations.

Patients aged ≥18 years who underwent ICL implantation at our institution were included in this study. The following were the inclusion criteria: (1) stable myopia (≤0.5 diopter [D] progression within 2 years); (2) ACD ≥2.6 mm (from the endothelium); (3) intraocular pressure <21 millimeters of mercury (mm Hg); and (4) endothelial cell density ≥2500 cells/mm^2^. The exclusion criteria were as follows: (1) corneal dystrophy; (2) suspected corneal ectasia; (3) history of ocular surgery/trauma; or (4) history of other ocular/systemic disease.

According to the ACD cutoff in the European Union and the FDA, the eyes were divided into shallow (ACD <3.0 mm) and normal ACD groups (ACD >3.0 mm).^11^ All examinations were performed before surgery and at 3 months postoperatively.

### SS-OCT Assessment

SS-OCT was performed using the CASIA2 device (Tomey, Nagoya, Japan), which captures 128 cross-sectional anterior segment images at a wavelength of 1310 nm in the darkroom. The entire anterior segment structure was scanned by applying minimal pressure to the eyeball while carefully pulling the upper and lower eyelids up and down, respectively. After the patients focused on the internal target, a 360-degree global scan was performed for each eye.

**Figure 2.**
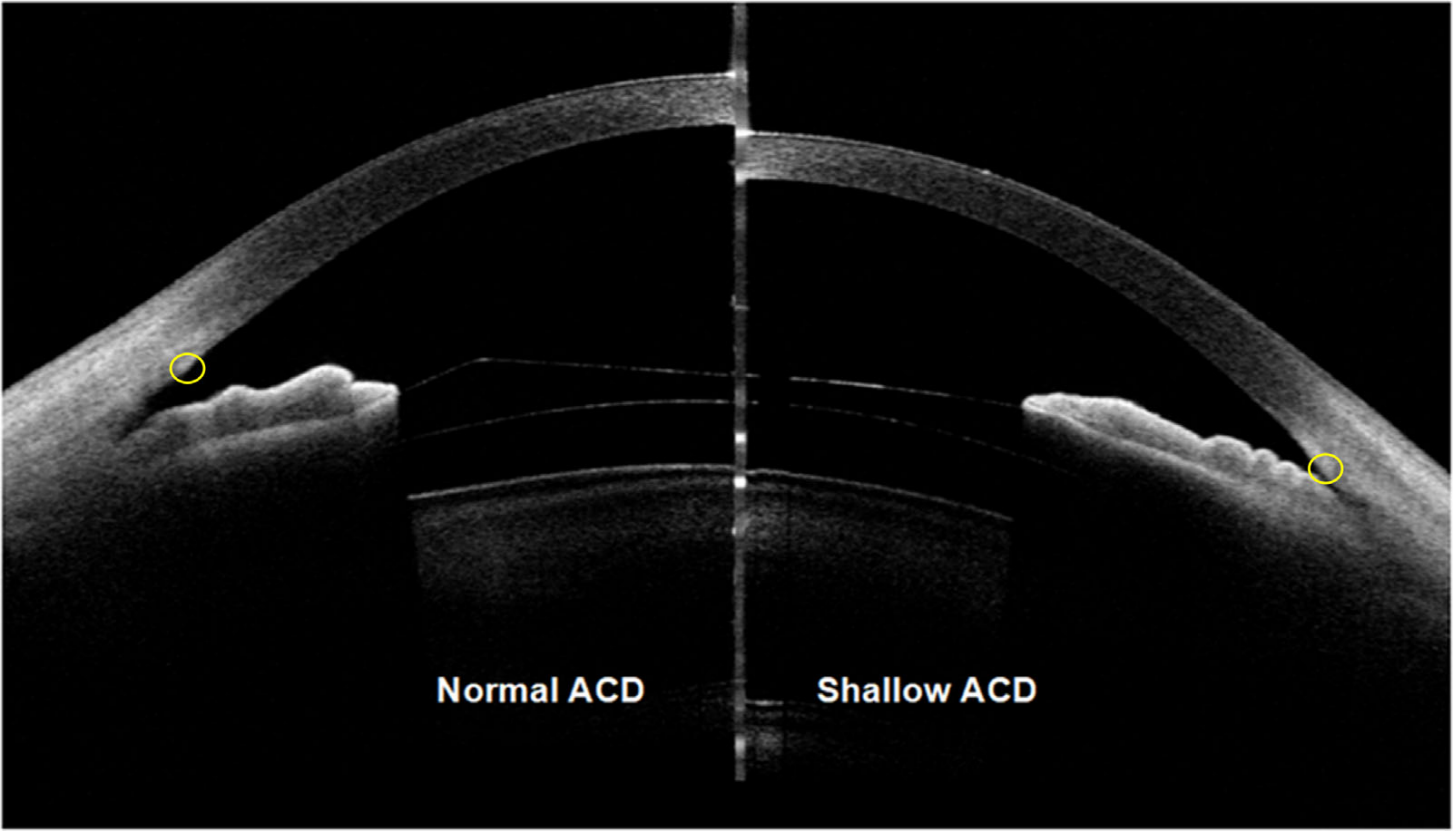
Representative two-dimensional anterior segment optical coherence tomography images of shallow and normal anterior chambers, scleral spur (*yellow ring*).

The position of the scleral spur (where the sclera protrudes inward, corresponding to the change in curvature of the corneoscleral junction; Fig. 2)^12^ was independently identified by two masked observers blinded to preoperative/postoperative status. In our previous study, we have conducted consistency analyses within and between observers according to this method (intraclass correlation coefficient [ICC] = 0.95, 95% confidence interval [CI] = 0.91–0.97).^13^ To ensure blinding, all postoperative images were randomized and analyzed without clinical metadata (e.g. surgery date or preoperative biometrics). The device software automatically calculated the anterior segment parameters, including local—trabecular-iris angle 500 µm from the scleral spur (TIA_500_), angle opening distance 500 µm from the scleral spur (AOD_500_), and trabecular-iris space area 500 µm from the scleral spur (TISA_500_—and 360-degree circumferential parameters—compositive area of the entire circumferential distance from AOD_500_ (AODA_500_), compositive volume of the entire circumferential area from TISA_500_ (TICV_500_), and ACV. For sector ACA analysis, the parameters obtained for the left eyes were reflected in the vertical axis, so that the nasal/temporal characteristics of the right and left eyes could be combined.

### Surgical Treatment

Before the surgery, ICL size and power were determined based on the white-to-white distance, sulcus-to-sulcus distance, and ACD, and modified vertex formulas calculations (http://en.informer.com/iclpower-calculation-software/, version 3.0), respectively**.** All surgical procedures were performed by an experienced surgeon (author X.Z.) and all patients received ICL V4c, as previously described.^14^ Postoperative medications included topical 0.1% prednisolone acetate 4 times a day for 4 days, topical 0.5% levofloxacin 4 times a day for 7 days, and topical pranoprofen 4 times a day for 14 days.

### Statistical Analysis

The Shapiro-Wilk test was used to assess the normality of data distribution. Pre- and postoperative ACA parameters were compared using paired *t*-tests, whereas between-group comparisons were made using independent samples *t*-tests. Pearson's and Spearman's correlation analyses were performed for normally and abnormally distributed data, respectively. Statistical significance was set at *P* < 0.05.

## Results

### Patients’ Characteristics

A total of 120 eyes of 120 patients (77% female patients) were enrolled in this study, 60 in each group. All eye surgeries were uneventful, and no postoperative surgery-related adverse events were observed. The mean preoperative manifest spherical equivalent was −8.34 ± 2.53 D (range = −15.75 to −2.50 D) in the shallow ACD group, and −7.98 ± 2.58 D (range = −13.00 to −1.75 D) in the normal ACD group (*P* = 0.449, independent samples *t*-tests). Three months postoperatively, the mean vault size was 0.495 ± 0.212 mm and 0.509 ± 0.204 mm in the shallow and normal ACD groups, respectively (*P* = 0.536; see Fig. 2). Patients’ demographic data are presented in Table 1.

**Table 1.** The Represented Demographic Data for the Patients

Characteristic	Shallow ACD Group	Normal ACD Group	*P* Value
Patients eyes, *n*	60, 60	60, 60	–
Age, y	28.6 ± 5.2 (22 to 47)	27.8 ± 5.2 (19 to 43)	0.445
Sex, male/female	10/50	18/42	–
Refractive errors, D			
Spherical	−8.05 ± 2.49 (−15.00 to −2.25)	−7.68 ± 2.51 (−12.50 to −1.75)	0.429
Cylindrical	−0.58 ± 0.51 (−1.50 to 0.00)	−0.60 ± 0.54 (−1.50 to 0.00)	0.899
MRSE	−8.34 ± 2.53 (−15.75 to −2.50)	−7.98 ± 2.58 (−13.00 to −1.75)	0.449
Corneal front Km, D	43.17 ± 1.40 (40.40 to 46.50)	43.31 ± 1.62 (38.10 to 46.70)	0.626
WTW, mm	11.61 ± 0.29 (11.1 to 12.5)	11.70 ± 0.35 (11.0 to 12.6)	0.295
ACD, mm	2.86 ± 0.07 (2.66 to 2.98)	3.26 ± 0.13 (3.06 to 3.59)	<0.001^*^
PD, mm	6.56 ± 0.81 (3.93 to 7.82)	6.85 ± 0.82 (4.61 to 8.53)	0.052
IOP, mm Hg	14.5 ± 2.49 (11.5 to 20.0)	14.4 ± 3.06 (11.0 to 21.5)	0.387
ICL size, mm	12.80 ± 0.34 (12.1 to 13.2)	12.97 ± 0.54 (12.1 to 13.2)	0.439
Post-vault, mm	0.495 ± 0.212 (0.198 to 1.057)	0.509 ± 0.204 (0.131 to 0.916)	0.536
Post-PD, mm	6.36 ± 0.75 (3.59 to 7.67)	6.48 ± 0.91 (4.13 to 8.08)	0.154

ACD, anterior chamber depth; D, diopters; ICL, implantable collamer lens; IOP, intraocular pressure; Km, mean keratometry; MRSE, manifest refraction spherical equivalent; PD, pupil distance; WTW, white to white.

* Note: The *P* value represents statistical significance.

### ACA Analysis

ACA parameters are presented in Table 2. Relative to the preoperative values, the TIA_500_, AOD_500_, and TISA_500_ values decreased significantly in both groups (all *P* < 0.01, paired *t*-tests). In the shallow ACD group, the values decreased from 48.008 ± 8.766 degrees, 0.547 ± 0.139 mm, and 0.185 ± 0.049 mm^2^ to 24.788 ± 4.226 degrees, 0.251 ± 0.048 mm, and 0.095 ± 0.018 mm^2^, respectively. In the normal ACD group, the respective values decreased from 59.338 ± 9.671 degrees, 0.766 ± 0.201 mm, and 0.266 ± 0.076 mm^2^ to 28.355 ± 4.441 degrees, 0.291 ± 0.051 mm, and 0.110 ± 0.018 mm^2^, respectively. Additionally, AODA_500_, TICV_500_, and ACV values decreased by 52.1%, 48.2%, and 38.9%, respectively, in the shallow ACD group (all *P* < 0.001), and by 59.2%, 56.6%, and 37.4%, respectively, in the normal ACD group (all *P* < 0.001). Notably, the reduction in all ACA parameters in the shallow ACD group was significantly smaller than that in the normal ACD group (all *P* ≤ 0.001).

**Table 2.** Changes in ACA Parameters

	Shallow ACD Group			Normal ACD Group					
	Pre	Post	Δ	Pre	Post	Δ	*P* Value*	*P* Value*#*	*P* Value
TIA_500_, degree	48.008 ± 8.766	24.788 ± 4.226^*^	23.220 ± 8.376	59.338 ± 9.671	28.355 ± 4.441^*^	30.983 ± 9.066	<0.001^*^	<0.001^*^	<0.001^*^
AOD_500_, mm	0.547 ± 0.139	0.251 ± 0.048^*^	0.296 ± 0.131	0.766 ± 0.201	0.291 ± 0.051^*^	0.475 ± 0.181	<0.001^*^	<0.001^*^	<0.001^*^
TISA_500_, mm^2^	0.185 ± 0.049	0.095 ± 0.018^*^	0.090 ± 0.046	0.266 ± 0.076	0.110 ± 0.018^*^	0.156 ± 0.068	<0.001^*^	<0.001^*^	<0.001^*^
AODA_500_, mm^2^	17.996 ± 4.275	8.332 ± 1.640^*^	9.664 ± 3.995	25.351 ± 6.270	9.876 ± 1.728^*^	15.475 ± 5.610	<0.001^*^	<0.001^*^	<0.001^*^
TICV_500_, mm^3^	6.169 ± 1.602	3.070 ± 0.755^*^	3.099 ± 1.551	8.937 ± 2.473	3.665 ± 0.780^*^	5.272 ± 2.221	<0.001^*^	<0.001^*^	<0.001^*^
ACV, mm^3^	170.068 ± 14.965	103.541 ± 11.979^*^	66.528 ± 15.092	203.888 ± 22.846	127.439 ± 18.740^*^	76.449 ± 17.668	<0.001^*^	<0.001^*^	0.001^*^
ITC index, %	0	0.390 ± 1.611	–	0	0.185 ± 0.814	–	–	–	–
ITC area, mm^2^	0	0.017 ± 0.08	–	0	0.007 ± 0.032	–	–	–	–

* *P* value: Postoperative parameters versus preoperative parameters in the shallow ACD group.

# *P* value: Postoperative parameters versus preoperative parameters in the normal ACD group.

*P* value: The reduction of ACA parameters in the shallow ACD group versus the reduction of ACA parameters in the normal ACD group.

### Sector ACA Analysis

**Figure 3.**
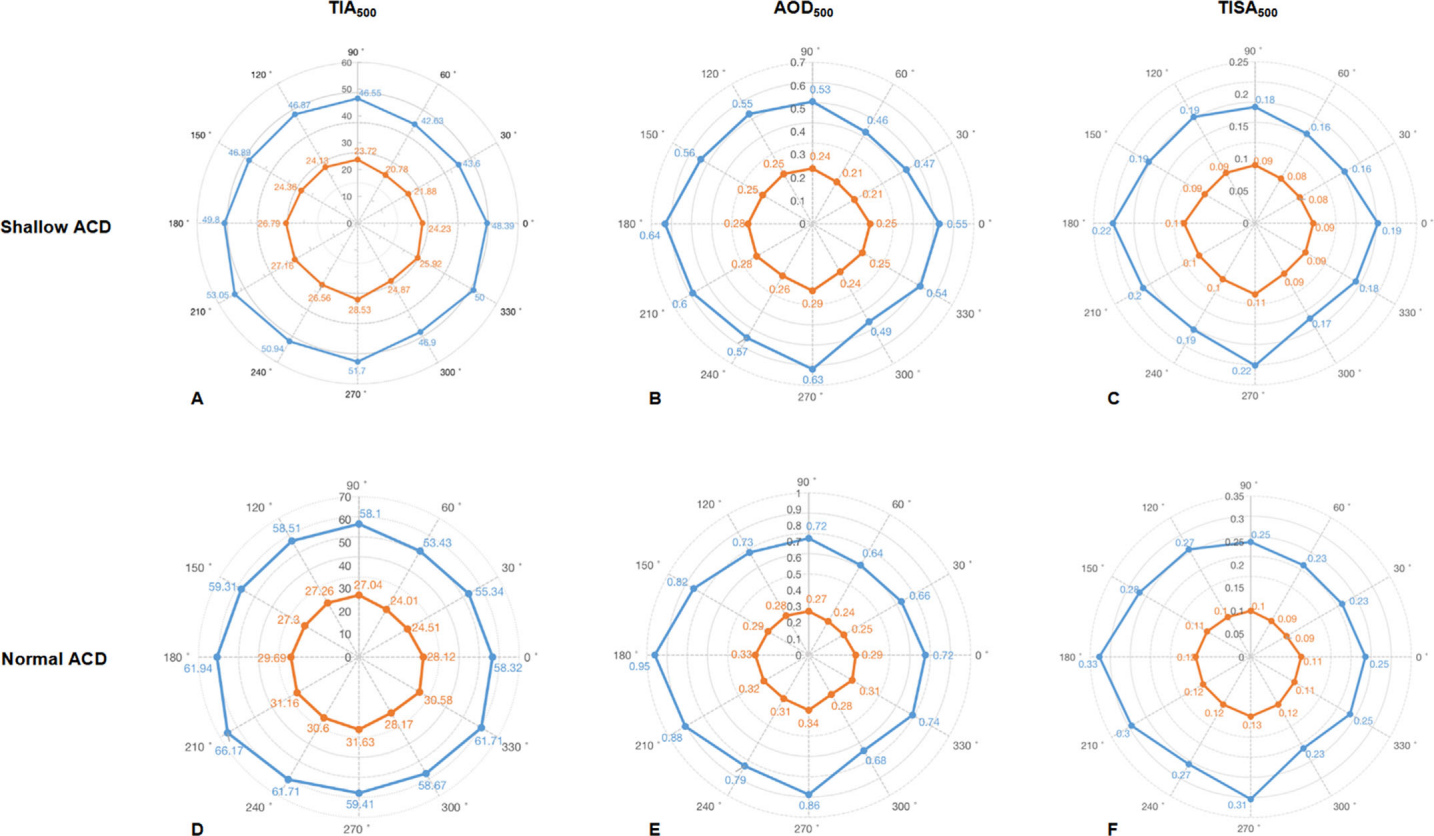
Circumferential 360-degree anterior chamber angle changes in shallow (TIA_500_) (**A**); AOD_500_ (**B**); TISA_500_ (**C**); and normal anterior chambers (TIA_500_) (**D**); AOD_500_ (**E**); TISA_500_ (**F**). *Blue line* = preoperative parameters, *orange line* = postoperative parameters.

Figure 3 shows the ACA parameters in each meridian before and after surgery. Preoperative TIA_500_, AOD_500_, and TISA_500_ values indicated that the ACA was narrowest in the superior-nasal quadrant and widest in the inferior-temporal quadrant for both groups. After surgery, for both groups, the distribution of TIA_500_, AOD_500_, and TISA_500_ values showed a similar tendency as the preoperative values. No significant difference in ACA parameters changes was observed among the 12 meridians in each eye. Notably, the decrease in ACA parameters in each meridian was greater in the normal ACD group than in the shallow ACD group.

### Subgroup Analysis

Vault was categorized as low (<500 µm) or high (≥500 µm) based on the midpoint of the manufacturer-recommended optimal range (250–750 µm).^15^^,^^16^ The ACD, vault size, and ACA parameter changes according to the presence of low or high vault in the small and normal ACD groups are presented in Table 3. There were significant differences between the small and normal ACD groups in ACD parameters for both subgroups (all *P* < 0.001), and the vault size in the high vault subgroup was significantly greater than in the low vault subgroup for both groups (all *P* < 0.001). At postoperative 3 months, the reduction in ACA parameters was more significant in the normal ACD group than in the shallow ACD group for both low (all *P* ≤ 0.005) and high vaults (all *P* ≤ 0.004). Additionally, ACA parameter reduction was significantly greater in the high vault subgroup than in the low vault subgroup for both groups (all *P* ≤ 0.035). The correlation analyses examining the relationships between vault size and ACA parameter changes indicated that vault size was positively correlated with ΔTIA_500_, ΔAOD_500_, ΔTISA_500_, ΔAODA_500_, ΔTICV_500_, and ΔACV in both groups (all *P* < 0.05, Pearson's and Spearman's test; Fig. 4).

**Table 3.** Changes in ACA Parameters

	Shallow ACD Group			Normal ACD Group				
	Low Vault	High Vault	P Value	Low Vault	High Vault	P Value	P Value*	P Value#
ACD, mm	2.847 ± 0.070	2.877 ± 0.076	0.124	3.229 ± 0.112	3.294 ± 0.144	0.05	<0.001*	<0.001*
Vault, mm	0.311 ± 0.065	0.680 ± 0.128	<0.001*	0.333 ± 0.090	0.684 ± 0.113	<0.001*	0.277	0.887
ΔTIA_500_, degree	18.580 ± 6.350	27.860 ± 7.606	<0.001*	27.643 ± 8.245	34.323 ± 8.726	0.005*	<0.001*	0.003*
ΔAOD_500_, mm	0.227 ± 0.084	0.364 ± 0.134	<0.001*	0.418 ± 0.180	0.533 ± 0.167	0.013*	<0.001*	<0.001*
ΔTISA_500_, mm^2^	0.067 ± 0.026	0.114 ± 0.051	<0.001*	0.138 ± 0.071	0.174 ± 0.060	0.035*	<0.001*	<0.001*
ΔAODA_500_, mm^2^	7.542 ± 2.619	11.786 ± 4.037	<0.001*	13.651 ± 5.634	17.299 ± 5.040	0.011*	<0.001*	<0.001*
ΔTICV_500_, mm^3^	2.285 ± 0.840	3.913 ± 1.679	<0.001*	4.623 ± 2.313	5.921 ± 1.952	0.022*	<0.001*	<0.001*
ΔACV, mm^3^	56.325 ± 12.408	76.730 ± 9.699	<0.001*	66.383 ± 14.081	86.515 ± 15.073	<0.001*	0.005*	0.004*
ΔITC index, %	0.157 ± 0.858	0.623 ± 2.106	–	0.120 ± 0.657	0.250 ± 0.952	–	–	–
ΔITC area, mm^2^	0.008 ± 0.043	0.025 ± 0.952	–	0.004 ± 0.019	0.011 ± 0.042	–	–	–

* *P* value: Changes of ACA parameters in the shallow ACD group versus changes of ACA parameters in the normal ACD group in low vault.

# *P* value: Changes of ACA parameters in the shallow ACD group versus changes of ACA parameters in the normal ACD group in high vault.

**Figure 4.**
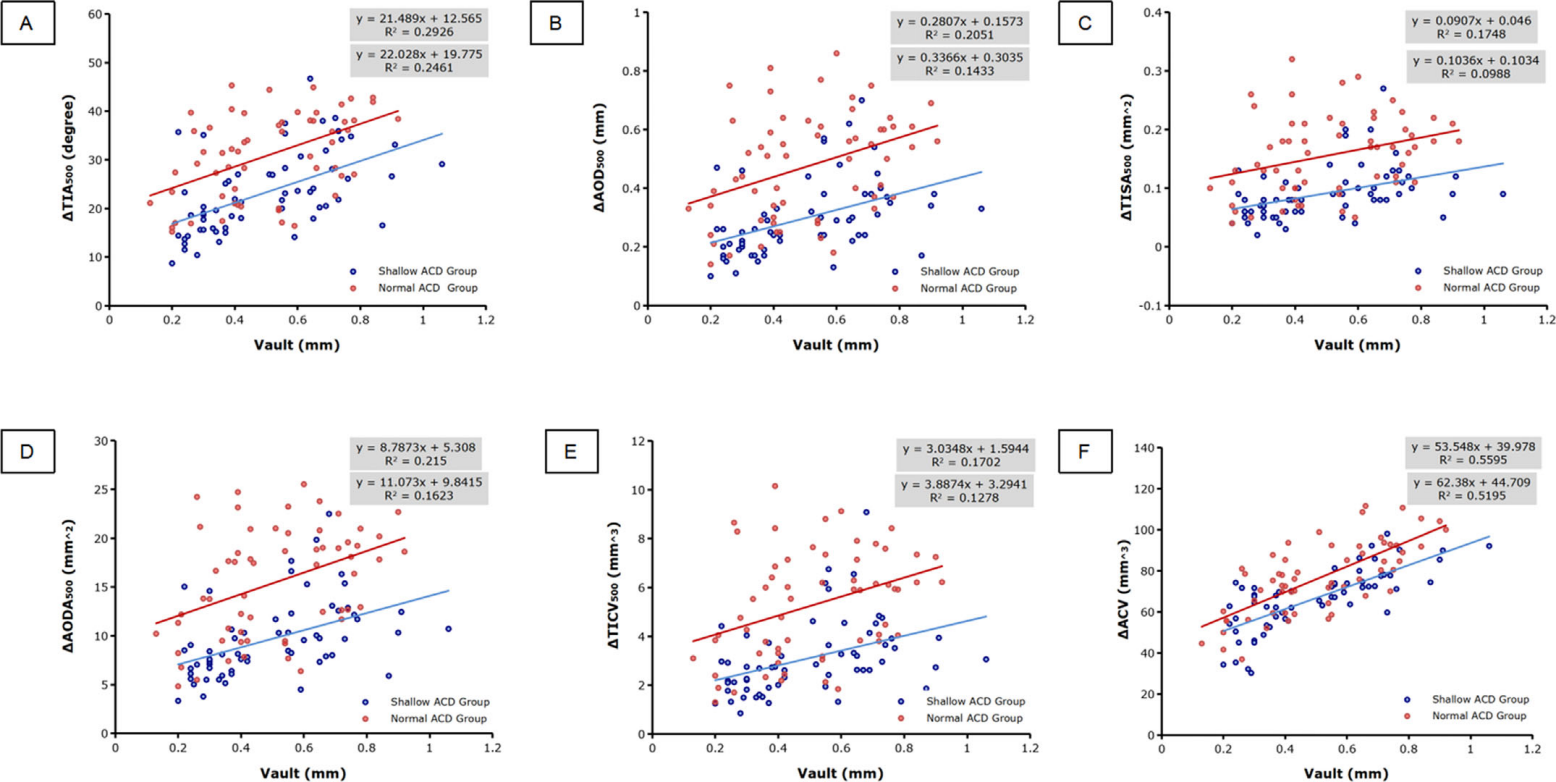
Correlation analysis of vault size and anterior chamber angle changes (**A–F** represent the relationship between vault size and the changes of TIA_500_, AOD_500_, TISA_500_, AODA, TICV, and ACV, respectively).

## Discussion

In this study, we found that all ACA parameters significantly decreased after ICL implantation in both groups relative to the preoperative values, for both low and high vaults. Notably, the reduction in the shallow ACD group was significantly shallower than that in the normal ACD group. Furthermore, the ACA was narrowest in the superior-nasal quadrant and widest in the inferior-temporal quadrant for both groups, both pre- and postoperatively. Additionally, vault size was positively correlated with all ACA parameter changes.

In ICL surgery, prediction of the postoperative ACA is vital for the prevention of complications related to secondary anterior chamber stenosis, particularly for patients with shallow ACD.^7^^,^^8^ In the past, postoperative ACA assessment was mostly based on local quadrant or single meridian images. In this study, 3-dimensional detailed data were obtained by automatic 360-degree global SS-OCT scanning, which could comprehensively evaluate ACA changes. The postoperative reduction in ACA parameters observed in our study, including TIA_500_ (47.3% vs. 51.3%), AOD_500_ (52.2% vs. 60.0%), TISA_500_ (46.6% vs. 56.1%), AODA_500_ (52.1% vs. 59.2%), TICV_500_ (48.2% vs. 56.6%), and ACV (38.9% vs. 37.4%), was significantly greater in the normal ACD group than in the shallow ACD group. Fernandez et al. reported that TIA_500_, AOD_500_, and TISA_500_ decreased by 34.5% to 42%, 50.3% to 58.4%, and 46% to 59.2%, respectively, at 3 months after ICL surgery.^7^ Based on ultrasound biomicroscopy (UBM) examinations, Chung and Lim et al. found that TIA_500_ and AOD_500_ decreased by 31.76% (38.1 ± 8.7 vs. 26.0 ± 6.5) and 41.45% (517.2 ± 180.2 vs. 302.8 ± 90.2 µm), respectively, in normal ACD eyes, and by 28.41% (31.92 ± 6.78 vs. 23.27 ± 8.14) and 27.32% (370.56 ± 105.27 vs. 265.27 ± 112.83 µm), respectively, in shallow ACD eyes 1 month after ICL implantation.^3^^,^^5^ These differences may be due to the differences in equipment and patient race: our SS-OCT-based study revealed greater postoperative angle reductions (TIA500/AOD500: shallow ACD 48.4%/54.1%, normal ACD 52.2%/62.0%) than Chung and Lim's UBM findings (31.76%/41.45% in normal, and 28.41%/27.32% in shallow ACD), which likely reflects both SS-OCT’s superior resolution (5 µm vs. 50 µm) capturing subtle anatomic changes and inherent racial disparities. Prior research indicates Chinese eyes have a naturally narrower ACA compared to Caucasian eyes (ACD/anterior chamber width [ACW] = 2.31 ± 0.24/11.52 ± 0.38 mm vs. 2.48 ± 0.28/12.06 ± 0.34 mm), even after ACD adjustments.^17^^,^^18^

An ACA of grade ≤2 (−20 degrees) is more likely to cause peripheral anterior adhesions and angular closure.^19^^–^^21^ Therefore, the postoperative ACA is a key anatomic parameter in determining the risk of primary angle-closure glaucoma. In this study, the preoperative TIA was greater than 20 degrees in all eyes. Notably, during the 3-month follow-up period, no patients developed glaucoma or exhibited sustained IOP elevation (>21 mm Hg on 2 consecutive visits), despite 7 eyes (11.7%) in the shallow ACD group and 1 eye (1.7%) in the normal ACD group demonstrating postoperative TIA <20 degrees (Shaffer grade 2). It can be concluded that, although the decrease in ACA in patients with shallow anterior chamber is smaller than that in patients with normal ACD, the postoperative ACA was worth our more attention due to the small preoperative ACA.

Prior studies have suggested that preoperative anterior chamber parameters (ACD, ACA, and ACV) are the main predictors of postoperative ACA values, and the vault size is considered to be the main factor causing ACA changes.^7^^,^^8^^,^^22^^,^^23^ Fernandez et al. showed a positive correlation between pre- and postoperative TIA values based on OCT results.^8^ Zhao et al. showed through stepwise multiple regression analysis using Pentacam examination that the preoperative central ACD was significantly correlated with the postoperative peripheral ACD, and the preoperative ACA was significantly correlated with the postoperative ACA.^19^ Therefore, the postoperative ACA was narrower in patients with a shallow anterior chamber, suggesting that preoperative ocular parameters could predict changes in ACA parameters.^7^^,^^8^ We also found that ACA changes were smaller in the shallow ACD group regardless of vault size. In addition, the subgroup analyses showed that ACA changes were greater in eyes with a high vault in both the shallow and normal ACD groups. Moreover, the correlation analysis showed that ACA changes were significantly correlated with the vault size. Previous studies have also found correlations between postoperative ACA and vault size, despite the use of different devices.^7^^,^^8^^,^^21^^,^^24^ Therefore, vault size was identified as an important factor influencing postoperative ACA values, suggesting that a higher vault may shift the ICL forward and lead to ACA narrowing. Some authors have proposed that ACA narrowing after ICL surgery is caused by a tent effect mediated by the ICL. It may be due to the protruding anterior surface of the ICL, which causes a 360-degree displacement of the iris in the pupil region by tent effect. Garcia-De et al. found that the iridocorneal angle increased with decrease in the vault even in photopic conditions.^25^ However, other studies found no significant correlation between vault size and postoperative ACA values, which may be due to the small distribution range of the vault size affecting the correlation analysis.^26^ Additionally, a larger ICL caused a higher vault, which also resulted in a greater change in ACA values after surgery.^22^

In order to understand the changes of the postoperative circumferential ACA in detail, we further studied the ACA parameters from 128 meridian scan images. The results showed that the distribution of ACA parameters before and after ICL surgery showed a similar trend, with the ACA being widest in the inferior-temporal quadrant and narrowest in the superior-nasal quadrant. Therefore, it can be inferred that the inherent anatomic variability of the ACA still plays a role under the influence of external factors. In addition, we found no significant differences in ACA changes across all meridians in both the shallow and normal ACD groups. Fernandez et al. observed a similar degree of ACA narrowing in the horizontal and inferior quadrants.^7^^,^^8^ These results are evidence that ICL surgery is reducing the ACA parameters in different quadrants by the same tenting effect.

This study has some limitations. Our observation period was at 3 months postoperatively; thus, long-term ACA changes and their influencing factors could not be elucidated. In addition, previous studies^27^ have demonstrated that both ICL size and IOP play significant roles in predicting postoperative ACA changes. However, ICL size selection is predominantly guided by the ciliary sulcus diameter, whereas IOP typically stabilizes and returns to baseline levels following surgery. Although the manual white-to-white (WTW) measurement offers clinical accessibility, UBM provides superior anatomical accuracy for ciliary sulcus visualization in ICL positioning, although its reproducibility is limited by operator dependency, whereas SS-OCT achieves optimal consistency in angle assessment.^28^ Current sizing algorithms (manufacturer's nomogram, and NK formula and KS formula) lack a gold standard; however, Asian-specific validation studies prioritize the NK formula for patients with a shallow ACD to achieve optimal vault outcomes.^16^^,^^29^ Although lens rotation was not required for vault adjustment in this study, its efficacy in angle widening and vault optimization has been validated in prior studies.^30^ In the future study, we will also refer to the research^31^ of deep learning technology on postoperative arch height to further explore the prediction effect of eye anatomic parameters on postoperative ACA value. Despite these limitations, to the best of our knowledge, this is the first study to observe circumferential ACA changes after ICL surgery in patients with a shallow anterior chamber.

In summary, we found that although eyes with a shallow anterior chamber exhibited smaller ACA changes than those in eyes with a normal anterior chamber, the postoperative ACA was smaller and associated with vault size in these eyes. Therefore, anterior segment structure was more crowded in patients with a shallow ACD. It is recommended to implement a longitudinal monitoring protocol that includes IOP tracking, angular endoscopy, and quarterly scans using nasal quadrant mapping on SS-OCT, particularly for high vaults cases (>750 µm). This comprehensive approach aims to identify and manage risks associated with progressive angular remodeling that could lead to glaucoma development. Future studies should develop a predictive model of the postoperative ACA to prevent complications associated with surgical secondary chamber angle stenosis.
